# Solving the maize maze? Look to the lower leaves

**DOI:** 10.1093/plphys/kiae236

**Published:** 2024-04-26

**Authors:** Ritu Singh

**Affiliations:** Assistant Features Editor, Plant Physiology, American Society of Plant Biologists; Department of Plant Science, University of California, Davis, CA, 95616, USA

As the global population edges toward 9 billion by 2050, a 60% to 100% estimated increase in crop production is crucial (Prajal et al. 2015; Tian et al. 2021). Among the key crops targeted for enhanced productivity is maize (*Zea mays* L.), a staple worldwide and presently the most widely cultivated cereal crop. However, urbanization, economic shifts, and increased meat consumption have significantly encroached on and fragmented arable lands. Given the constraints of diminishing arable land for maize cultivation, optimizing maize production per unit area becomes paramount for ensuring food security (Xin et al. 2023; Zhang et al. 2023). Planting density emerges as a critical agronomic factor in maize production, with studies indicating that increased planting density can boost maize yield by an average of 17% to 20% (Assefa et al. 2018; Luo et al. 2023). This approach facilitates efficient utilization of available sunlight, thereby enhancing the conversion of carbon dioxide and water into grains (Ma et al. 2020). Nonetheless, dense planting can produce intra-specific competition for essential resources, such as light in aerial tissues and nutrients and water in roots, potentially reducing per-plant growth and yield (Deng et al. 2012; Yan et al. 2021; Duan et al. 2023).

In this issue of Plant Physiology, Yan et al. (2024) investigated the mechanisms associated with maize single-plant yield loss under high-density planting. Over 2 years, they conducted field experiments in northwestern China using 3 maize varieties, MC670, LY66, and JK968, at 4 planting densities between 75,000 and 135,000 plants/hectare. Additionally, to analyze canopy profiles, the authors vertically categorized the aerial parts of the plants into 4 layers, I to IV, based on ear positioning (Fig.). Statistical analysis revealed significant effects of planting density and maize varieties on population scale maize yield, with no notable interaction between density and variety. Under dense planting conditions, total yield peaked at 105,000 plants/ha for each variety, with MC670 exhibiting the most significant increase, followed by LY66 and JK968. This increase in yield under high-planting density was primarily related to an increase in ear number per unit area, countering decreases in kernel number per plant and kernel weight. Moreover, higher planting density also increased the barren stalk rate, particularly in the JK968 followed by LY66 and MC670. Furthermore, at increased planting density, the population-level biomass also increased, especially in layer II of the canopy, with the JK968 showing the smallest increment compared to LY66 and MC670.

**Figure.**
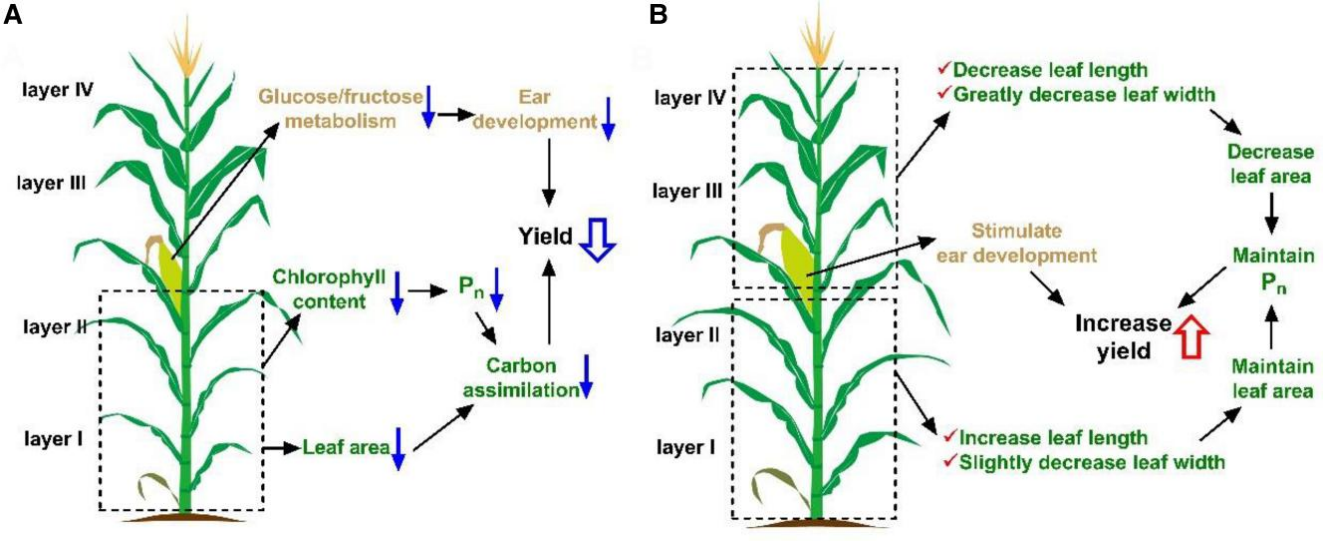
Schematic diagram illustrating the physiological mechanisms behind yield fluctuations in plants at varying planting densities (adapted from Yan et al. (2024), Figure 9). **A)** Depicts reduced grain yield under high-density planting, attributed to diminished photosynthetic rates and leaf area in the lower canopy layers, leading to decreased assimilate accumulation. Additionally, dense planting density impacts glucose and fructose levels in young ears, hindering ear development, subsequently diminishing kernel number per ear and per-plant kernel weight, resulting in per-plant yield loss. **B)** Highlights the potential for increased yield under optimized planting density conditions through optimizing leaf morphology in canopy layers. This will enhance the photosynthetic rates and ear development, thus higher yield. Blue and red arrows signify decreases and increases, respectively, in the highlighted processes. Yellow, green, and black denote ear development, carbon metabolism, and yield, respectively.

To understand the dynamics at the individual plant level, the authors assessed yield parameters. The results indicate that high planting density reduced per-plant yield, particularly in the JK968 variety. Increased planting density also caused pronounced decreases in per-plant biomass accumulation across cultivars and planting years. Significant reductions in biomass accumulation in the lower canopy (layers I and II) were observed in JK968, followed by LY66, then MC670. Biomass accumulation, both at population and single-plant levels, particularly in the lower canopy, was positively correlated with grain yield and grain weight per plant. Moreover, more total biomass transfer was observed in layers I and II than in layers III and IV. Overall, biomass transfer was greatest in MC670 and lowest in LY66. These findings suggest biomass accumulation and transport in the lower canopy are important for yield formation under high-density planting conditions.

Photoassimilation is the fundamental basis of plant productivity and biomass production, with leaves being the primary organs responsible for light interception and photosynthesis (Gaju et al. 2016; Chen et al. 2019). To investigate the impact of planting density on photosynthesis, Yan and coworkers analyzed the total leaf area, width, and length of individual leaves at each canopy layer. In LY66 and MC670 varieties, leaf area and length gradually increased with leaf position in the lower canopy of the plant, peaked in the middle, and then gradually decreased towards the upper canopy. Additionally, leaf width is reduced in the upper canopy. Conversely, JK968 exhibited an opposite trend compared to these 2 varieties. Increased planting density increased the spatial density of leaf area across all 4 canopy layers, implying weaker canopy transmission and poor light quality under high-density conditions. As a result of low light interception, the net photosynthetic rate (Pn) and chlorophyll content decreased in high density. However, the decrease in Pn and total chlorophyll content of MC670 was relatively smaller in the lower canopy layers than the other varieties, which can be attributed to its leaf pattern. Additionally, the red-to-far red ratio decreased in the lower canopies of LY66 and JK968 plants but not in MC670. Overall, the observed leaf area patterns allowed more photosynthetically active radiation to reach the lower layers in MC670 followed LY66, resulting in higher photosynthetic rates and increased chlorophyll contents in layers I and II of these plants than in JK968. Alternatively, it can be inferred that the light quality was superior in the lower canopy layers of MC670 compared to LY66 or JK968.

Kernel number is an important yield parameter and is also affected by photosynthetic capacity and photoassimilate accumulation. The authors observed that ear length and diameter decreased along with increased planting density, whereas the bald tip length increased. Among the varieties, JK968 was the most sensitive to increased planting density with respect to the increase of bald tip length. Moreover, higher planting density resulted in plant growth delays, specifically during the silking stage, whereas the tassel was minimally affected, resulting in the extended anthesis-silking interval (ASI). ASI causes asynchronous flowering, hindering successful pollination and leading to yield losses of ∼40% to 50% (Uribelarrea et al. 2008; Sher et al. 2017). Moreover, increasing planting density increased starch content in the ears but reduced levels of soluble sugars, especially glucose and fructose. Thus, increased planting density may have hindered ear metabolism, inhibiting development and yield. Overall, ear length, glucose levels, and fructose levels were most strongly impacted by planting density in JK968 plants, followed by MC670, then LY66.

In conclusion, the results indicated that high planting density led to decreased photosynthetic capacity of leaves within the lower canopy, resulting in reduced biomass production. Furthermore, increased planting density suppressed ear development, causing significant per-plant yield loss. Additionally, the researchers suggested that reducing leaf length and width in the upper canopy could facilitate light penetration into the lower canopy, thereby enhancing photosynthesis in the lower canopy with increased leaf length and slightly decreased leaf width (Fig.). Consequently, strategies to maximize grain yield under high-density planting conditions should focus on 2 main areas: optimizing canopy structure to maintain high photosynthetic efficiency in the lower-canopy leaves and promoting ear development.

## Data Availability

No data is generated in this study.
